# Blood Lymphocyte Subsets for Early Identification of Non-Remission to TNF Inhibitors in Rheumatoid Arthritis

**DOI:** 10.3389/fimmu.2020.01913

**Published:** 2020-08-27

**Authors:** Eulalia Rodríguez-Martín, Israel Nieto-Gañán, Borja Hernández-Breijo, Cristina Sobrino, Carlota García-Hoz, Javier Bachiller, Ana Martínez-Feito, Victoria Navarro-Compán, Paloma Lapuente-Suanzes, Gema Bonilla, Dora Pascual-Salcedo, Garbiñe Roy, Teresa Jurado, Pilar Nozal, Mónica Vázquez-Díaz, Alejandro Balsa, Luisa M. Villar, Chamaida Plasencia-Rodríguez

**Affiliations:** ^1^Department of Immunology, IRYCIS, Hospital Universitario Ramón y Cajal, Madrid, Spain; ^2^Immuno-Rheumatology Research Group, IdiPaz Hospital Universitario La Paz, Madrid, Spain; ^3^Department of Rheumatology, IRYCIS, Hospital Universitario Ramón y Cajal, Madrid, Spain

**Keywords:** rheumatoid arthritis, TNF inhibitors, biomarkers, lymphocyte subsets, remission

## Abstract

**Background:** TNF inhibitors (TNFis) are widely used for the treatment of rheumatoid arthritis (RA), although the response rates to this therapy in patients with RA remains heterogeneous and < 50% achieve remission (REM).

**Objective:** To analyze baseline peripheral blood leukocytes profiles in order to search for biomarkers identifying patients who will most likely not achieve REM under TNFi treatment.

**Methods:** A prospective bi-center pilot study including 98 RA patients treated with TNFis and followed-up during 6 months. Patients were classified according to DAS28 as follows: those who achieved REM (DAS28 ≤ 2.6) and those who did not (DAS28 > 2.6) at 6 months after starting TNFis. These rates were also assessed by simplified disease activity index (SDAI ≤ 3.3 and SDAI > 3.3, respectively). Peripheral blood immune cells were studied by flow cytometry before treatment initiation.

**Results:** At 6 months, 61 or 80% of patients did not achieve REM by DAS28 or SDAI, respectively. Basal leukocyte profiles differed between REM vs. non-REM patients. Non-REM patients showed lower percentages of total and naïve B cells at baseline than REM subjects. A B lymphocyte/CD4+ lymphocyte ratio (BL/CD4 ratio) <0.2 clearly associated with a higher probability of non-REM status based on DAS28 at 6 months (OR = 9.2, *p* = 0.006). These data were confirmed when patient response was evaluated by SDAI index.

**Conclusion:** Our results strongly suggest that BL/CD4 ratio could be considered as a useful biomarker for the early identification of non-remitters to TNFi in clinical practice.

## Introduction

Rheumatoid arthritis (RA) is a chronic, inflammatory and heterogeneous autoimmune disorder of unknown etiology characterized by progressive joint damage (1, 2). Different immune cells and effector pathways are involved in the cascade of events leading to the progression and persistence of this disease (3, 4). In recent years, a better understanding of the underlying immunopathogenic mechanisms has led to the development of targeted synthetic and potential biologic disease-modifying antirheumatic drugs (bDMARDs) for the treatment of the RA (5, 6). There are various treatments for RA depending on the severity of the disease and a given patient's response to a particular drug. Tumor necrosis factor inhibitors (TNFis) were the first developed as a subset of bDMARDs and have dramatically changed the therapeutic prerogatives for RA patients (7, 8). Nowadays, TNFis remain among the primary biological therapeutic options for patients with RA. TNF influences several pathologic processes, including joint destruction, and synovial hyperplasia (9), and promotes B-cell proliferation and immunoglobulin secretion (10).

Although TNFis have helped alter the natural history of RA, ~20–40% of patients do not respond to this therapy and around only 50% achieve remission (11, 12). Thus, the identification of biomarkers predicting clinical response at the beginning of a treatment would greatly aid therapeutic decisions. According to EULAR recommendations, remission (REM) or a state of at least low disease activity is the clinical target for RA patients (5). Although early treatment can prevent disabilities in many patients, the most effective new drugs are expensive. Therefore, the ability to determine the profile of patient who might benefit from such treatments is of the utmost importance in clinical settings. Several biomarkers have been proposed as effect predictors of REM in patients with RA. However, no robust biomarkers are currently available that can predict responses to different DMARDs (13, 14). Several immune cells are strongly associated with RA, and each cell type contributes in a different way to disease pathogenesis (15). Although RA has long been regarded as a T-cell-centered disorder, recent evidence suggests that B cells play an important role in the onset and perpetuation of this disease (16).

The main aim of the present study was to investigate whether the blood immunological profile of RA patients starting TNFis could aid in identifying those less likely to achieve REM. We studied the percentages and total cell counts of different peripheral blood immune cell subsets before initiating TNFis, which are known to assist in the regulation of B-cell homeostasis (17, 18).

## Patients and Methods

### Study Design and Population

This study was approved by the ethics committees of Ramón y Cajal (PI-018/17) and La Paz University Hospitals (PI-2618), Madrid, in accordance with the Helsinki Declaration. Each patient signed a written consent before enrollment. A code was assigned to every patient to warrant confidentiality of collected data. This was a prospective observational, longitudinal, bi-center pilot study that included 98 consecutive patients diagnosed with RA according to the 1987 revised criteria of the American Colleague of Rheumatology (19). All patients were undergoing TNFi treatment at Ramón y Cajal Hospital (*n* = 34) and La Paz Hospital (*n* = 64) between 2016 and 2018.

A TNFi treatment was initiated because of the presence of active disease (DAS28 > 3.2) despite prior treatment with synthetic DMARDs or a previous TNFi (*n* = 12 patients). Patients who had previously received rituximab, abatacept or tocilizumab were not included in this study. The cohorts of both hospitals were matched according to baseline demographic characteristics and treatments received to ensure that they were homogeneous populations.

### Evaluation and Follow-Up

Patients were included consecutively in the study and followed for 6 months. The primary endpoint was not achieving REM after 6 months of treatment as determined by a disease activity score 28 (DAS28); patients were classified as REM (DAS28 ≤ 2.6) or non-REM (DAS28 > 2.6). As a secondary clinical endpoint, clinical non-REM based on the simplified disease activity index (SDAI), was collected at the same time point, using SDAI ≤ 3.3 to classify patients as REM and SDAI > 3.3 as non-REM.

### Flow Cytometry Studies

Heparinized whole blood was collected from each patient immediately before initiating TNFis. Peripheral blood mononuclear cells (PBMCs) were obtained within 2 h by Ficoll-Isopaque density gradient centrifugation (Life Technologies Ltd, UK) and stored in liquid nitrogen in aliquots of 5–6 × 10^6^ cells until studied.

The following monoclonal antibodies were used in the blood lymphocyte subsets study: CD4-FITC, CD8-APC-H7, CD27-APC, CD14-FITC, CD197-PE (CCR7-PE), CD3-PerCP, CD3-BV421, CD19-PE-Cy7, CD45RO-APC, CD56-APC, CD45-V500-C (all from BD Biosciences, San Diego, CA).

**Figure 1:**
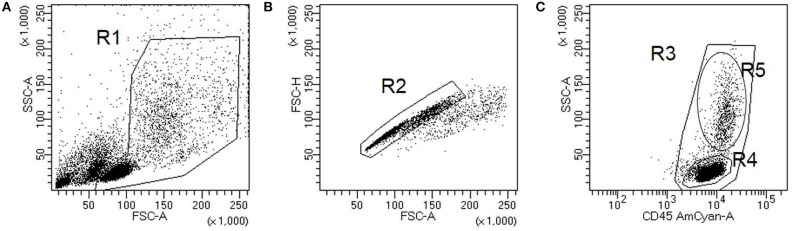
Representative images of gating strategy for flow cytometry analysis. Cells were first gated to exclude debris and apoptotic cells (**A**, gate R1). Single cells (**B**, gate R2) were further analyzed for their CD45 staining to identify peripheral blood mononuclear cells (PBMCs) (**C**, gate R3), lymphocytes (**C**, gate R4), and monocytes (**C**, gate R5).

**Figure 2:**
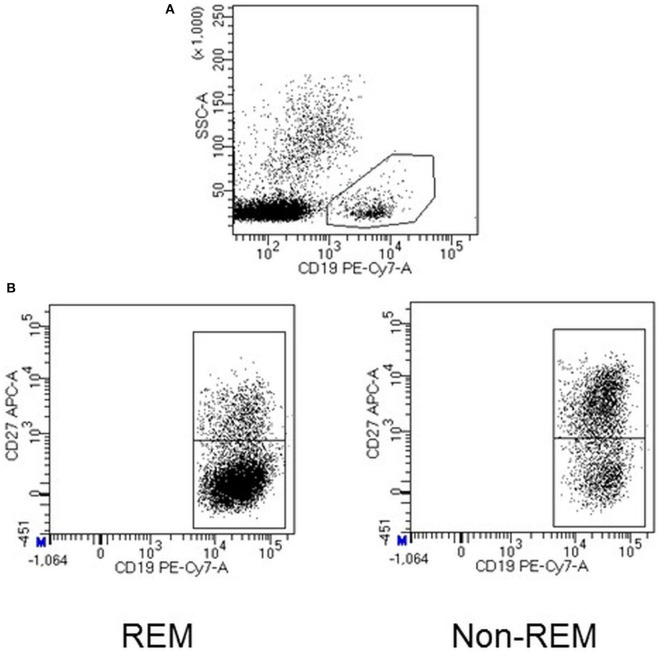
Representative dot plots showing total B cells, which were identified based on the expression of the cell surface marker CD19 **(A)**. B naïve cells from a patient who achieved remission (REM) after 6 months of TNFi therapy and one who did not (non-REM) **(B)**.

Blood leukocyte subsets were studied by flow cytometry as previously described (20). Briefly, for membrane antigen staining, 10^6^ PMBCs were labeled with the appropriate amounts of monoclonal antibodies during 30 min at 4°C in the dark, washed with PBS, and acquired in a FACSCanto II flow cytometer (BD Biosciences). Data analysis was performed with FACSDIVA software V.8.0 (BD Biosciences). A gate including lymphocytes and monocytes, but excluding debris and apoptotic cells, was established; a minimum amount of 100,000 events were analyzed. Representative images of the gating strategy used for the flow cytometry analysis are shown in Figure 1. Mean auto fluorescence values were set using appropriate negative isotype controls. According to the differential expression of several antigens, CD4+ and CD8+ T cells were classified as: naïve (CCR7+ CD45RO–); central memory (CM) (CCR7+ CD45RO+); effector memory (EM) (CCR7–CD45RO+); terminally differentiated (TD) (CCR7–CD45RO–). B cells were classified as: total CD19+ B cells, memory (CD19+CD27+), and naïve (CD19+ CD27–) (see Figures 2A–C); CD56+ cells were subdivided into natural killer (NK) cells (CD56dim CD3–), NKT cells (CD56dim CD3+), and NK regulatory cells (CD3–CD56bright).

### Statistical Analysis

Analyses were performed using the Statistical Package for the Social Sciences (SPSS 24, Chicago, IL, USA). Graphs were made using GraphPad Prism 6.0 software (GraphPad Prism Inc., La Jolla, CA, USA). We used Fisher's exact test for categorical variables, and the Mann–Whitney *U-*test for continuous ones. A *p*-value below 0.05 was considered statistically significant. To demonstrate that the significance between the different cellular subpopulations and clinical outcomes was not due to chance, the correction of multiple comparisons using the Benjamini-Hochberg (BH) method with a predefined value FDR = 0.25 was applied (free software used MEV 2.0). Cell populations significantly associated with our clinical outcome and this association with the pathogenesis of RA was used to define a baseline ratio to predict non-REM after 6 months of TNFi therapy. To confirm that the chosen ratio was the most appropriate, we calculated the Spearman's correlation with different basal cell ratios and the clinical activity measured by DAS28 at 6 months. After this, a ROC was performed to obtain the cut-off value associated with our clinical outcome. This cut off was chosen by using the Youden Index. We used our clinical outcome defined by DAS28 because this is the activity index most frequently employed in clinical practice for patients with RA. Later, it was verified that this cut-off point was also useful for another activity score, namely SDAI. Univariate and multivariate analysis were carried out to investigate which factors were independently associated with a lower probability of achieving REM. Univariable analysis was carried out using the following variables: age, sex, concomitant methotrexate, baseline prednisone dose, baseline activity score (DAS28 or SDAI), baseline C reactive protein (CRP), previous TNFi, disease duration, rheumatoid factor (RF) and anti-citrullinated antibodies (ACPA), body mass index and smoking habit. In the multivariate analysis, only the variables with a *p* < 0.1 in the univariable analysis were included.

### Patient and Public Involvement

Patients did not cooperate with us in the design of the study. However, it was explained to them prior to inclusion and they all agreed to participate.

## Results

### Patient Characteristics

Ninety-eight patients (85% female) who underwent TNFi therapy for at least 6 months were consecutively included in the study. Baseline demographic and clinical data of REM and non-REM patients based on DAS28 are shown in Table 1.

**Table 1:** Baseline characteristics of patients treated with TNFis (*n* = 98).

**Clinical and epidemiological variables**	**Total patients** **(*n =* 98)**	**Non-REM** **DAS28 > 2.6** **(*n =* 60; 61%)**	**REM** **DAS28 ≤ 2.6** **(*n =* 38; 39%)**	***p*-value**
**Sex (female)**	83 (85)	54 (90)	29 (76)	0.1
**Age (years)**	53 ± 13	54 ± 14	52 ± 12	0.5
**Disease duration (years)**	8 (4–12)	8 (4–12)	7 (4–11)	0.9
**RF positive**	76 (78)	42 (71)	34 (90)	**<0.05**
**ACPA positive**	82 (84)	46 (78)	36 (95)	**<0.05**
**CRP (mg/L)**	5.7 (2.1–12.1)	6.4 (2.5–18.3)	3.0 (0.7–9.6)	**<0.01**
**Smoking habit**				
No	38 (46)	26 (53)	12 (36)	0.1
Yes	44 (54)	23 (47)	21 (63)	
**Body mass index (kg/m^2^)**	24.8 (22.9–29.7)	25.4 (23.1–29.6)	24.7 (22.0–30.2)	0.3
**DAS28**	4.9 ± 1.1	5.3 ± 1.0	4.3 ± 0.9	**<0.001**
**Previous TNFi treatment**	12 (12)	7 (12)	5 (13)	1.0
**TNFi type**				
Monoclonal antibodies	53 (54)	37 (62)	16 (42)	0.1
Etanercept	45 (46)	23 (38)	22 (58)	0.1
**TNFi monotherapy**	3 (3)	1 (2)	2 (5)	1.0
**Concomitant DMARDs**	95 (97)	59 (98)	36 (95)	1.0
Only OD	21 (21)	11 (19)	10 (27)	0.2
MTX ± OD	74 (76)	48 (79)	26 (68)	1.0
**Prednisone use**	57 (59)	36 (60)	21 (55)	0.8

*The table shows mean ± standard deviation (SD), median (IQR) or absolute number (percentage) for all included patients (n = 98). REM: patients who achieved remission after 6 months of TNFi therapy; Non-REM: patients who did not achieve remission; RF, rheumatoid factor; ACPA, anti-citrullinated protein antibodies; DAS28, Disease Activity Score-28; CRP, C reactive protein; TNFis, Tumor Necrosis Factor inhibitors; csDMARD, conventional synthetic disease-modifying anti-rheumatic drug; MTX, methotrexate; OD, other conventional synthetic disease-modifying anti-rheumatic drugs than methotrexate. The differences between DAS28 groups were analyzed considering p < 0.05 as statistically significant result. Bold values mean statistically significant results*.

Most patients were biological naïve (88%, *n* = 86) and only 12 had previously received a TNFi. This heterogeneity was considered in the interpretation of the results.

An analysis was performed excluding the twelve patients that previously received a TNFi and no significant differences were observed when compared with results obtained with the whole cohort (98 patients). Different TNFis were used in the study, mainly etanercept (46% of patients), whereas 54% received monoclonal antibodies (infliximab, adalimumab, certolizumab, or golimumab). Only 3% of patients underwent TNFi monotherapy.

A total of 38 (39%) and 60 patients (61%) were in REM and non-REM based on DAS28, respectively, 6 months after starting a TNFi. Non-REM patients were more frequently negative for RF (71 vs. 90%; *p* = 0.033) and ACPA (78 vs. 95%; *p* = 0.026), and had higher CRP levels [6.4, [2.5–18.3] vs. 3.0, [0.7–9.6] mg/l; median (Mdn), interquartile range [IQR]; *p* = 0.004] and higher DAS28 (5.3 ± 1.0 vs. 4.3 ± 0.9; *p* < 0.001) at baseline. REM based on SDAI occurred in 23 (20%) patients.

### Leukocyte Subsets at Baseline in Patients Classified According to DAS28 After 6 Months of TNFis Initiation

The percentages of B cells [4.0, [2.8–6.8] in non-REM vs. 5.8, [4.0–9.5] in REM] and naïve B cells [3.4, [2.0–5.4] in non-REM vs. 4.4, [3.1–6.5] in REM] were lower in non-REM compared to REM patients (Figures 2B,C). No significant differences were seen in the percentages of the other PBMC subsets (naïve, memory and effector CD4+ and CD8+ T cells subsets, monocytes, NK, NKT and NK regulatory cells) at baseline between REM and non-REM patients (Table 2).

**Table 2:** Percentages and absolute numbers of leukocyte blood subsets shown by REM and non-REM patients at baseline of TNFi therapy.

	**Percentages**			**Absolute numbers**		
**Effector and memory subsets**	**Non-REM** ***n =* 60** **Mdn (IQR)**	**REM** ***n =* 38** **Mdn (IQR)**	***p***	**Non-REM** ***n =* 60** **Mdn (IQR)**	**REM** ***n =* 38** **Mdn (IQR)**	***p***
**CD4+T cells**	46.3 (35.9–56.5)	43.5 (27.6–54.1)	0.2	**974 (686–1495)**	**763 (514–1046)**	**0.03**
Naïve	22.6 (10.1–33.6)	20.3 (7.6–30.7)	0.4	434 (210–800)	341 (131–617)	0.2
Central memory	14.2 (11.8–18.7)	14.1 (11.1–18.4)	0.7	323 (233–421)	293 (189–355)	0.1
Effector memory	3.6 (1.1–7.5)	5.1 (2.0–7.0)	0.8	72 (19–190)	98 (33–121)	0.3
Terminally differentiated	2.1 (0.8–4.3)	2.9 (0.6–5.3)	0.9	55 (15–106)	61 (10–107)	0.9
**CD8+T cells**	12.5 (8.7–17.4)	14.1 (10.5–18.0)	0.3	274 (180–437)	259 (175–392)	0.8
Naïve	3.8 (6.4–2.5)	2.6 (3.8–6.3)	0.6	89 (49–173)	67 (44–132)	0.4
Central memory	1.2 (0.8–1.9)	1.3 (0.8–1.8)	0.7	25 (17–39)	24 (12–38)	0.3
Effector memory	2.4 (1.2–5.1)	2.5 (1.3–4.2)	0.9	85 (44–161)	90 (51–122)	0.6
Terminally differentiated	3.0 (1.9–5.4)	3.0 (2.1–5.4)	0.3	56 (36–144)	65 (35–122)	0.6
**NKT cells**	3.6 (2.0–6.2)	4.4 (2.8–7.5)	0.9	79 (50–126)	73 (53–163)	0.7
**NK cells**	11.9 (7.3–16.2)	10.6 (6.0–16.8)	0.5	236 (157–364)	170 (116–365)	0.2
**NK regulatory cells**	0.7 (0.3–1.1)	0.6 (0.3–1.2)	0.9	15 (8–26)	13 (6–22)	0.4
**CD19+** **B cells**	**4.0 (2.8–6.8)**	**5.8 (4.0–9.5)**	**0.02**	84 (57–164)	111 (80–186)	0.1
Naïve B cells	**3.4 (2.0–5.4)**	**4.4 (3.1–6.5)**	**0.03**	67 (45–142)	86 (53–149)	0.1
Memory B cells	0.9 (0.6–1.3)	1.1 (0.7–2.1)	0.4	19 (11–37)	17 (14–36)	0.9
**Monocytes**	6.7 (3.8–10.6)	8.3 (5.1–13.1)	0.5	171 (77–265)	145 (88–261)	0.9

*Values are expressed as percentages of total peripheral blood mononuclear cells and as absolute numbers (cells/μl). REM: patients who reached remission after 6 months of TNFi therapy; Non-REM: patients who did not reach remission. The differences between DAS28 groups were analyzed considering p < 0.05 as statistically significant result. Mdn (IQR), median (interquartile range); NK, natural killer cells; NKT, natural killer T cells; NS, not significant; REM, remission. Bold values mean statistically significant results*.

Total basal cell numbers of CD4+ T cells were significant higher in non-REM [974, [686–1,495]] than in REM patients [763, [514–1,046]; *p* = 0.03]. In the other hand, B and B naïve cells tended to be lower in non-REM compared to REM patients [B cells: 84.3, 57.4–164.0 vs. 184.1, 80.2–186.0, *p* = 0.063; B naïve cells: 67.3, [45.2–142.0] vs. 142.8, [53.3–149.3], *p* = 0.076]. No significant differences were observed in the absolute cell number of the rest of PBMC subsets at baseline. Results are shown in Table 2. After applying the multiple test correction Benjamini-Hochberg (B–H), the percentage of total B cells and B naïve cells remained significant (*p*-value added B–H 0.18 and 0.21, respectively). In addition, the probable effect of baseline prednisone use and dose on the percentages of total and naïve B cells as well as on the B/CD4 T cell ratio was analyzed without finding statistical signiffication (see Supplementary Material, Supplementary Table 2).

### Defining a Baseline B Lymphocytes/CD4+ T Cells (BL/CD4) Ratio Associated With Clinical Outcome

According to the data obtained we explored if ratios between B and CD4+ T cells at baseline could further identify patients with high probability of not reaching REM at 6 months. Two possible baseline ratios could be chosen: basal BL/CD4+ ratio or basal BnL/CD4+. We first explored the association of both ratios with clinical activity at 6 months of treatment measured by DAS28. Both gave modest positive correlations (Spearman's rho coefficient: −0.316, *p* = 0.003 for BL/CD4+ ratio and −0.296, *p* = 0.005 for BnL/CD4+ratio) (see Table 1 in Supplementary Material, Supplementary Table 1).

However, to confirm that the BL/CD4+ ratio was the most appropriate choice, a Spearman's correlation with other ratios combining other cell subpopulations involved in the pathogenesis of RA was calculated (Supplementary Table 1). Although several baseline ratios (BL/Tcells, BL/CD4+, BnL/CD4+, BL/CD8+) correlated significantly with the DAS28 at 6 months, the BL/CD4+ was the one that obtained the greatest correlation (Supplementary Table 1).

An ROC curve using a BL/CD4+ ratio was performed to find a cut-off value for predicting the probability of not achieving REM (AUC: 0.651, *p* = 0.015). Finally, a BL/CD4+ ratio < 0.2 was associated with a higher probability of not attaining REM at 6 months after treatment (sensitivity: 34%, specificity: 91%, negative predictive value: 70%). In the univariable analysis, being a female (OR: 2.8, 0.9–8.6, *p* = 0.074), negative for RF (OR: 3.4, 1.1–11.2, *p* = 0.04) and ACPA (OR: 5.0, 1.1–23.9, *p* = 0.04), having higher baseline DAS28 (OR: 3.0, 1.8–5.0, *p* < 0.001), higher baseline CRP (OR: 1.1, 1.01–1.1, *p* = 0.026) and a basal BL/CD4+ <0.2 (OR: 2.8, 1.1–7.7; *p* = 0.04) associated with not achieving REM based on DAS28 6 months after treatment (see Table 3). In the multivariable analysis, having a basal BL/CD4+ < 0.2 (OR: 9.2, 1.9–44.5; *p* < 0.006), a higher DAS28 (OR: 3.9, 2.0–7.5; *p* < 0.0001) significantly associated with not achieving REM based on DAS28 6 months after starting TNFis (data shown in Table 3).

**Table 3:** Univariate and multivariate analyses associated with not achieving REM at 6 months after starting TNFi therapy.

**Univariate analysis**			
**Variable**	**OR**	**95% IC**	***p***
Female sex	2.8	0.9–8.6	0.074
Age at starting TNFi	1.01	0.9–1.04	0.550
BMI	1.04	0.9–1.1	0.352
Smoking habit	1.9	0.8–4.9	0.139
Disease duration	0.98	0.95–1.0	0.223
**Negative RF**	**3.4**	**1.1–11.2**	**0.040**
**Negative ACPA**	**5.1**	**1.1–24.0**	**0.040**
**Baseline DAS28**	**3.0**	**1.8–5.0**	**<0.0001**
**Baseline CRP**	**1.05**	**1.01–1.1**	**0.026**
Baseline Prednisone dose	0.9	0.8–1.0	0.240
Baseline MTX	1.7	0.7–4.4	0.276
Previous TNFi use	1.1	0.3–3.9	0.826
**Baseline BL/CD4+ <0.2 ratio**	**2.8**	**1.1–7.7**	**0.040**
**Multivariate analysis**			
**Variable**	**OR**	**95% IC**	***p***
Female sex	2.1	0.4–10.8	0.364
Negative RF	7.0	0.9–50.4	0.054
Negative ACPA	1.6	0.2–13.0	0.682
**Baseline DAS28**	**3.9**	**2.0–7.5**	**<0.0001**
Baseline CRP	1.0	0.9–1.1	0.193
**Baseline BL/CD4+ <0.2 ratio**	**9.2**	**1.9–44.5**	**0.006**

*Bold values mean statistically significant results*.

Furthermore, when using the SDAI definition to not achieve REM after 6 months of TNFi treatment, having a baseline BL/CD4+ ratio < 0.2 was the only variable independently associated with not attaining REM (OR: 4.2, 1.2–14.6; *p* = 0.026) in the multivariable analysis, was based on SDAI. This further show the value of this biomarker in predicting response to TNFi in patients with RA. This analysis was adjusted for the same confounder except baseline CRP levels because this variable is included in SDAI score.

## Discussion

In this study, we deepened into the leukocyte profiles of RA patients at baseline TNFis by studying a wider array of leukocytes, including different subtypes of T, B, and NK cells and monocytes in a cohort of 98 RA patients. We explored changes by comparing results obtained in REM vs. non-REM patients. Our results demonstrated that REM patients have a higher percentage of total B cells due to the presence of naïve B cells than non-REM patients. Furthermore, we found that a BL/CD4+ ratio < 0.2 prior to starting a TNFi was associated with a 9-fold greater probability of not achieving REM based on DAS28 and 4-fold greater based on SDAI.

Several blood biomarkers have been proposed as indicators of RA. RF and ACPA are autoantibodies locally produced in the inflamed synovium by B cells. Clinical usefulness of these autoantibodies as diagnostic and prognostic factors of disease are now widely accepted (21). Moreover, B cells mediate T-cell activation via the expression of costimulatory molecules and by secreting pro-inflammatory cytokines, particularly TNFα (22). However, none of these indicators proved useful for predicting clinical response to treatment. Our study showed that 39% of patients with RA achieved remission based on DAS28 and 20% according to SDAI after 6 months of TNFi therapy. At baseline, REM patients had lower CRP levels and DAS28 and were more frequently positive for RF and ACPA than non-REM patients. Whereas, being RF seropositive correlated with a better clinical response to biologics such as rituximab and abatacept (23), this issue was regarded as too controversial in relation to TNFis. At present, neither RF nor ACPA can be reliably used as absolute markers of prognosis and response to this treatment (24).

Several lines of evidence have pointed to B-cell function as a critical factor in the development of RA (16). Disturbances in B-cell development have been detected in RA, as well as in other autoimmune conditions such as systemic sclerosis, Sjogren's syndrome and systemic lupus erythematous (25–27). A decrease in circulating memory B cells and a predominance of CD27-naïve B cells have been reported in these autoimmune diseases, including the very early stages of RA and other polyarthritis (25). Other authors have reported that high levels of CD27+ memory B cells **>*26%*** at baseline correlated with good clinical response to TNFis (28). In this study, Daien et al. (28) evaluate 21 patients that received etanercept, certolizumab pegol, and adalimumab and were followed 3 months after TNFi initiation. These authors define response to treatment on the basis of EULAR criteria, a decrease in DAS28 score > 0.6 between baseline and 3 months, with a DAS28 score < 5.1 at 3 months. By using this criteria, EULAR responders at 3 months includes patients with mild to moderate response to TNFi. On the other hand, Annolik et al. (29) describe the effect of etanercept in the decrease of CD27+ memory B cells and support the notion that anti-TNF therapy may impair B-cell functionality via its effects on follicular dendritic cells and by disrupting germinal center formation and maintenance. According to these results, elimination of these structures in the synovial microenvironment could potentially disrupt tissue-specific pathogenic B-cell activation. The increase of naïve B cells in REM patients described in the present study is in agreement with several of these studies and may be due to the fact that during the initial phase of RA, memory B cells are recruited not only to the synovial membrane, but also to secondary lymphoid organs where they become activated. Our 98 patients were followed for 6 months and response to TNFi was defined on the basis of DAS28 ≤ 2.6. This criteria includes as REM patients only those with a strong response, excluding mild to moderate responders. Differences in study designs, response to treatment criteria, patient characteristics and smaller sample sizes, could explain conflicting results (18).

The most important therapeutic target in RA is to eliminate signs and symptoms and to prevent joint damage, thereby halting disease progression and lessening physical function disability (1). This goal can be made possible by achieving REM, when disease activity ceases or only residual disease activity remains. The proportion of patients achieving REM varies according to the scores used. In this study, we selected DAS28 to define REM because it is the composite score most widely used in clinical practice. In addition, we also analyzed our proposed BL/CD4+ ratio predicting REM by SDAI, obtaining similar results. The increasing number of bDMARDs, the heterogeneous responses to these treatments (11, 12) and the relevance of obtaining adequate clinical outcomes all demand the continued search for biomarkers that can better predict the best line of therapy for patients with RA. Measuring the baseline BL/CD4 ratio is easy to perform and could be a useful tool for making more effective and personalized decisions for patients with RA before embarking on a TNFi treatment.

One of the limitations of this study could be that the sample size is not very large. However, before starting the patient recruitment, in the calculation of the sample size, 98 patients were enough to demonstrate statistical differences in the proposed clinical outcomes. This study was performed using consecutively recruited RA patients who had started TNFi treatment at two different hospitals. The sample size and clinical characteristics of the pooled patients were similar to those of other RA cohorts previously published in the literature. No differences in the response rate depending on the TNFi used were previously reported by other authors. Our study design included an examination of the main immune cell blood subsets. A very interesting aspect worth pursuing in future investigations is whether or not patients who fall below the defined ratio in this study and who do not achieve REM with TNFis are more likely to achieve the same clinical outcome when they undergo treatment involving a different mechanism of action.

Our future research projects, following this line of work, includes to confirm the consistency of these results in an independent cohort to evaluate if these cell subpopulations are modified after the administration of TNFi treatment, and studying whether the basal PBMC analysis may be useful to predict response to biological drugs with different mechanism of action (anti-IL6 receptor, CTLA4, and anti-CD20).

In summary, our results suggest that basal B naïve percentages and a BL/CD4 ratio may help identify which patients with RA are more likely that not to achieve REM before using TNFi. Specifically, a ratio of BL/CD4+ ratio < 0.2 prior to starting TNFis was associated with a 9-fold higher probability of not achieving REM based on DAS28 and 4-fold higher according to the SDAI index. Further investigations should be undertaken to obtain a better understanding of the roles that these cell subpopulations (BL and CD4+) play in the pathology of this disease.

## Data Availability Statement

All datasets generated for this study are included in the article/Supplementary Material.

## Ethics Statement

The studies involving human participants were reviewed and approved by Ethics committees of Ramón y Cajal (PI-018/17) and La Paz University Hospitals (PI-2618), Madrid. The patients/participants provided their written informed consent to participate in this study.

## Author Contributions

ER-M, DP-S, LV, and CP-R planned the study. IN-G, BH-B, CG-H, PL-S, and TJ collected samples and performed flow cytometry experiments. ER-M and CP-R wrote the manuscript draft. ER-M and LV supervised flow cytometry studies. BH-B, CP-R, and VN-C performed and supervised statistical studies. PN and GR performed RF, ACPA, and CRP quantification. CS, JB, MV-D, AB, GB, VN-C, and CP-R visited RA patients and collected clinical data. ER-M and CP-R had full access to all of the data in the study and take responsibility for the integrity of the data and the accuracy of the data analysis. All authors were involved in drafting the article or revising it critically for important intellectual content, and all authors approved the final version to be published. All authors contributed to the article and approved the submitted version.

## Conflict of Interest

AB reports grants, personal fees and other from Abbvie and Pfizer, grants and personal fees from Novartis, grants from BMS, personal fees and other from Nordic and Lilly, other from Sanofi, personal fees from Sandoz and UCB, during the conduct of the study. The remaining authors declare that the research was conducted in the absence of any commercial or financial relationships that could be construed as a potential conflict of interest.
