# Supplementary material for: Blood Lymphocyte Subsets for Early Identification of Non-Remission to TNF Inhibitors in Rheumatoid Arthritis
Source: Front Immunol. 2020 Aug 27;11:1913. doi: 10.3389/fimmu.2020.01913 (PMC7481468; doi:10.3389/fimmu.2020.01913)
Supplement: Supplementary file 1 [file Table_1.docx]

**Supplementary data**

**Table 1s**. Spearman´s correlation between baseline ratio of different cells subpopulations and clinical activity measured by DAS28 after 6 months of TNFi therapy.

|  | DAS28 at 6 months | |
| --- | --- | --- |
| Baseline ratio | Spearman´s rho coefficient | p- value |
| BL/T cells* | -0.287 | 0.007 |
| **BL/CD4+** | **-0.316** | **0.003** |
| BnL/CD4+* | -0.296 | 0.005 |
| BL/CD8+ | -0.246 | 0.021 |
| BL/Mono* | -0.120 | 0.266 |

*BL: B lymphocytes; BnL: B naïve lymphocytes; Mono: monocytes.
