# Supplementary material for: Blood Lymphocyte Subsets for Early Identification of Non-Remission to TNF Inhibitors in Rheumatoid Arthritis
Source: Front Immunol. 2020 Aug 27;11:1913. doi: 10.3389/fimmu.2020.01913 (PMC7481468; doi:10.3389/fimmu.2020.01913)
Supplement: Supplementary file 2 [file Table_2.docx]

**Table 2s.** Association between baseline prednisone (use and dose) and peripheral blood cell profile. Mann-Whitney U and Spearman correlation (r) analysis were respectively performed.

|  | non Prednisone use | Prednisone use | p-value |
| --- | --- | --- | --- |
| Total B cells (%) | 4.3 (3.1-7.8) | 5.2 (3.5-7.1) | 0.5 |
| Naïve B cells (%) | 3.3 (2.3-6.0) | 4.2 (2.8-6.4) | 0.3 |
| B/CD4 T cells ratio | 0.13 (0.07-0.21) | 0.10 (0.06-0.17) | 0.4 |

|  | Correlation Coefficient (r) | p-value |
| --- | --- | --- |
| Total B cells (%) | -0.06 | 0.6 |
| Naïve B cells (%) | 0.01 | 0.9 |
| B/CD4 T cells ratio | -0.06 | 0.6 |
